# Climate factors associated with help seeking of patient suffering from suicidal stress: The case of rainy days

**DOI:** 10.1192/j.eurpsy.2021.1875

**Published:** 2021-08-13

**Authors:** D. Hernández-Calle, M.F. Bravo-Ortiz

**Affiliations:** Psychiatry, Hospital Universitario La Paz, Madrid, Spain

**Keywords:** Suicide, Emergency Care

## Abstract

**Introduction:**

Suicidal phenomena help seeking depends on a broad array of factors, which include climatic variables. Our aim was to analyse the effect of precipitation with help seeking in patients suffering from suicidal behaviour

**Objectives:**

Our aim was to analyse the effect of precipitation with help seeking in patients suffering from suicidal behaviour

**Methods:**

Daily urgency visits from suicidal phenomena (including suicide attempt and ideation) were extracted from electronic medical records of Hospital Universitario La Paz from 1st January 2019 to 31st December 2019. Precipitation data (measured as accumulated litres per square meter) was obtained from a local climate station. Spearman correlation was estimated

**Results:**

The Spearman correlation coefficient was 0.04(p = 0.48)

**Conclusions:**

Precipitation did not influence help seeking for patients affected by suicidal phenomena

**Disclosure:**

No significant relationships.

